# Dietary Intervention on Overweight and Obesity after Confinement by COVID-19

**DOI:** 10.3390/nu15040912

**Published:** 2023-02-11

**Authors:** José Ignacio Ramírez-Manent, Pilar Tomás-Gil, Pau Martí-Lliteras, Josep Lluis Coll Villalonga, Emilio Martínez-Almoyna Rifá, Ángel Arturo López-González

**Affiliations:** 1 Faculty of Medicine, University of the Balearic Islands, 07009 Palma, Spain; 2 IDISBA, Balearic Islands Health Research Institute Foundation, 07004 Palma, Spain; 3 General Practitioner Department, Balearic Islands Health Service, 07003 Palma, Spain; 4 Investigation Group ADEMA SALUD IUNICS, 07003 Palma, Spain; 5 Faculty of Dentistry, University School ADEMA, 07009 Palma, Spain

**Keywords:** index cardiometabolic, Mediterranean diet, mobile SMS, COVID-19, obesity

## Abstract

Background: Obesity has become a public health problem in our society and is associated with many diseases, including type 2 diabetes mellitus, cardiovascular diseases, dyslipidemia, respiratory diseases, and cancer. Several studies relate weight loss in obese patients to improved anthropometric measurements and cardiometabolic risk. The objective of our study was to evaluate anthropometric changes, analytical parameters, insulin resistance, fatty liver, and metabolic scales, after a personalized weight loss program, through dietary advice to increase adherence to the Mediterranean diet and a motivational booster via mobile SMS messaging. Methods: Intervention study on a sample of 1964 workers, in which different anthropometric parameters were evaluated before and after dietary intervention: the metabolic score of insulin resistance; non-alcoholic fatty liver disease using different scales; metabolic syndrome; atherogenic dyslipidemia; and the cardiometabolic index. A descriptive analysis of the categorical variables was performed, by calculating the frequency and distribution of the responses for each one. For quantitative variables, the mean and standard deviation were calculated, since they followed a normal distribution. Bivariate association analysis was performed by applying the chi-squared test (corrected by Fisher’s exact statistic when conditions required it) and Student’s *t*-test for independent samples (for comparison of means). Results: The population subjected to the Mediterranean diet improved in all the variables evaluated at 12 months of follow-up and compliance with the diet. Conclusions: Dietary advice on a Mediterranean diet and its reinforcement with reminder messages through the use of mobile phones may be useful to improve the parameters evaluated in this study and reduce the cardiometabolic risk of patients.

## 1. Introduction

Obesity has become a public health problem in both developed and developing countries, since it forms the basis of many other associated diseases. In the 20th century, the WHO warned of this problem—which it called a 21st-century pandemic—and recommended that a series of appropriate measures be taken to avoid and prevent its growth [1].

The World Health Organization (WHO) defines obesity as a chronic disease, characterized by increased body fat, associated with increased health risk [2]. According to the WHO, the fourth cause of risk of death worldwide in 2019, measured in all age groups and both sexes, was obesity [3].

This excessive fat accumulation increases the risk of other diseases, including type 2 diabetes mellitus, cardiovascular disease, dyslipidemia, respiratory disease, and cancer [4,5]. The increase in this pathology produces greater mortality, permanent disability, and loss of quality of life in people who suffer from it. This also has repercussions at the societal and health system level, with lower productivity at work, days lost at work due to illness, and increased costs terms of healthcare due to admissions secondary to pathologies triggered by obesity and the pharmacological costs they entail [6,7].

A current lifestyle with reduced or lack of physical exercise, together with the tendency to eat elaborate meals rich in saturated fat and carbohydrates, along with low consumption of fruits and vegetables, constitutes a very important risk factor for obesity [8]. These unhealthy lifestyle habits are already established in the child population and continue into adolescence, influenced by the eating behavior of the parents [9], which produces an adult society with greater obesity and, therefore, with a greater risk of developing other pathologies [10,11].

On 11 March 2020, the WHO declared a new disease caused by a new virus called SARS-CoV-2 [12], which was internationally known as “coronavirus disease 2019” as a global pandemic [13]. This situation led the governments of different countries to declare a state of confinement for several months to try to stop the transmission of infection [14]. In Spain, the Royal Decree 463/2020, of 14 March, declared a state of emergency [15], which was maintained until 21 June 2020. During this period of confinement, the population worsened their lifestyles, with a decrease in physical exercise and unhealthy eating, which led, among other things, to an increase in weight [16].

Diet is one of the measures used to lose weight [17,18]. There really is no single “ideal” diet. There are many possible diets, capable of satisfying the nutritional needs of our body. Among them is the so-called Mediterranean diet, which has been shown to provide cardiovascular and cognitive benefits to the population [19,20]. Advice on the Mediterranean diet is included in the database of the national health system of the autonomous communities involved in the study, and in the occupational health services. Likewise, in the aforementioned computer programs, there are different diets calculated by nutritionists with different caloric content. This makes it possible to provide each person with a balanced diet to lose weight. In the same way and in the same documents, the web page of the Ministry of Health, Consumption and Social Welfare of Spain [21] provides information on healthy lifestyles. On this page, interested people find advice on how to make a purchase, how to cook food, how to eat, plan menus, information about food, etc.

In this century, new technologies have been successfully used to implement different measures to establish healthy lifestyle habits. The use of computers and mobile phones has been shown to be effective in weight loss programs [22,23,24,25].

Health interventions through mobile devices (mHealth interventions) facilitate and reinforce lifestyle changes [26]. The use of mobile devices allows for low-cost interventions. Previous studies show that short SMS text messages can be effective in weight loss [27,28,29] and lifestyle changes [30,31,32]. A systematic review on the use of new technologies in lifestyle modification for the prevention of diabetes obtained significant improvements in weight loss [33].

Several studies have linked weight loss in the obese to the improvement in different anthropometric measurements and cardiometabolic risk [34,35,36]. However, we are not aware of any study that has assessed insulin resistance, fatty liver, and changes in anthropometric parameters, and cardiometabolic scales after performing a weight loss intervention.

The objective of our study was to assess anthropometric changes, analytical parameters, insulin resistance, fatty liver, and metabolic scales after the establishment of a personalized weight loss program, through the establishment of dietary advice to increase adherence to the Mediterranean diet and a motivational reinforcement through mobile SMS messaging.

## 2. Materials and Methods

### 2.1. Participants

**Figure 1:**
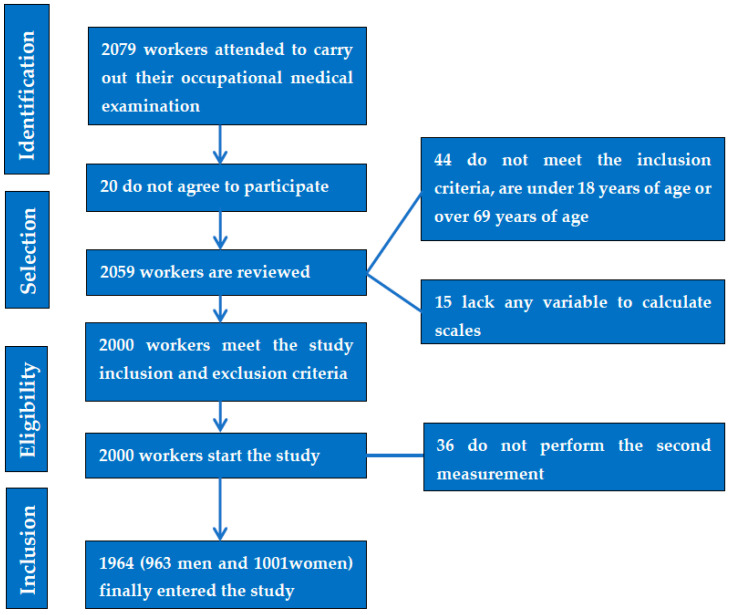
Prisma flow chart of the participants in the study.

An intervention study was conducted with a sample of 1964 workers from the autonomous communities of the Balearic Islands and Valencia (Spain), belonging to different professions. Patients were selected from those who attended an occupational medical examination between the months of January and June 2021, 6 months after the end of the confinement due to COVID-19 in our country. The inclusion criteria were: aged between 18 and 69 years; being healthy without illnesses that did not allow them to pass the medical check-up; giving their consent to participate in the study; and permission to use their data for epidemiological purposes. Figure 1 shows the flowchart of the participants in the study.

Social classes were classified according to the 2011 National Classification of Occupations (CNO-11), based on the proposal of the Spanish Society of Epidemiology, and divided into three categories: Class I: directors/managers, university professionals, athletes, and artists; Class II: intermediate occupations and self-employed workers without employees; Class III: unskilled workers [37].

A smoker was considered to be a person who had consumed at least one cigarette per day on a regular basis in the previous month or who had stopped smoking less than a year before.

Physical activity was determined by the International Physical Activity Questionnaire (IPAQ) [38], a self-administered questionnaire of seven questions that assesses the type of physical activity carried out in the previous 7 days.

Diet adherence was assessed using the Mediterranean diet adherence questionnaire [39], which includes 14 questions with values of 0 or 1 point each. A points total in response to the questions below 9 indicates low adherence, whereas above 9 indicates good adherence.

Anthropometric measurements, metabolic and analytical tests were performed at the beginning and end of the study in all participants. Anthropometric measurements and blood samples for analysis were carried out by health personnel from different occupational health units, after standardization of the techniques.

Weight (in kg) and height (in cm) were determined using a SECA 700 scale with an attached SECA 220 telescopic measuring rod, according to the international standards for ISAK anthropometric assessment [40]. Body mass index (BMI) was calculated by dividing weight by height in meters squared, and a patient was considered obese when their BMI was greater than 30 [41]. Waist circumference (WC) was measured with a SECA measuring tape with the person standing, feet together and trunk erect, with the abdomen relaxed. The tape was positioned parallel to the ground at the level of the last floating rib. A SECA model tape was used to measure hip circumference. The person stood in the previous position but, in this case, the tape measure was passed horizontally at hip height.

By dividing waist circumference by height and hip circumference, waist/height and waist/hip ratios were obtained. Cut-off points were 0.50 for the first index in both men and women and 0.85 for the second index in women and 0.95 in men [41].

Body fat percentage was determined by bioimpedance using a Tanita MC-780MA S model.

Blood pressure was measured with the person in the supine position after 10 min of rest. An OMRON M3 model calibrated automatic sphygmomanometer was used. Three determinations were made with 1-minute intervals between them, and the mean value was calculated.

Blood samples were obtained by puncture in a peripheral vein after 12 h of fasting, sent to the reference laboratories, and processed within 48–72 h of extraction. Glucose, cholesterol, and triglycerides (TG) were determined using automated enzymatic methods, and these results were expressed in mg/dL. HDL-cholesterol was determined by precipitation with dextran sulfate-MgCl2, while c-LDL was calculated using the Friedewald formula (providing TG were less than 400 mg/dL). Values were also expressed in mg/dL.

Determination of the percentage of the body’s insulin resistance was analyzed with the following scales:

The metabolic score for insulin resistance (METS-IR), which is a mathematical approach to quantify hepatic sensitivity to insulin using fasting parameters [42].

METS-IR = Ln [(2FPG) + TG] BMI/(Ln[HDLc])

The triglyceride glucose index (TyG), which is used for the presumptive diagnosis of insulin resistance [42].

TyG = Ln [fasting TG (mg/dL) FPG (mg/dL)/2]

The triglyceride glucose index–body mass index, which is a useful marker for insulin resistance in non-diabetic individuals (TyG-BMI) [42].

TyG-BMI = TyG BMI

The triglyceride glucose index–waist to height ratio (TyG-WtHR) [42].

TyG-WtHR = TyG WtHR

Triglyceride glucose index–waist circumference (TyC-WC) [42].

TyG-WC = TyG WC

SPISE-IR single-point insulin sensitivity index [43].

SPISE index (=600 × HDL0.185/Triglycerides0.2 × BMI1.338)

Scales to determine non-alcoholic fatty liver disease:

Lipid accumulation product (LAP) [42],

accumulation product (LAP) [42].

Men: LAP = (waist circumference (cm) − 65) (triglyceride concentration (mMol))

Women: LAP = (waist circumference (cm) − 58) (triglyceride concentration (mMol))

Fatty liver index (FLI) [42].

FLI = (log (triglycerides)100.953 + 0.139BMI + 0.71log (ggt) + 0.053 waist circumference − 15.745)/(1 + log (triglycerides) 100.953 + 0.139 BMI + 0.718 log (ggt) + 0.053 waist circumference − 15.745) 100

Hepatic steatosis index (HSI) [42].

HSI = 8 ALT/AST + BMI (+2 if type 2 diabetes yes, +2 if female)

Fatty liver disease index (FLD) [44].

BMI + Triglycerides + 3 × (ALT/AST) + 2 × Hyperglycemia (presence = 1; absence = 0)

Zhejiang University index (ZJU) [45].

BMI + Glycaemia (mmol L) + Triglycerides (mmol L) + 3ALT/AST + 2 if female

The BAAT [BMI, Age, ALT, TG] score was proposed as a clinical scoring system based on simple clinical or laboratory indices to identify advanced fibrosis in patients with non-alcoholic fatty liver disease (NAFLD) [46].

Waist-to-hip ratio (WthipR) was calculated by waist circumference (WC) divided by hip circumference (HC) [47].

Metabolic syndrome was assessed with three formulas:

(a) NCEP ATP III (National Cholesterol Education Program Adult Treatment Panel III) [48]. Metabolic syndrome is defined when at least three of the following factors are present: waist circumference greater than 88 cm in women and 102 in men; triglycerides with values higher than 150 mg/dL or if the person is receiving lipid-lowering treatment for this condition; blood pressure in figures greater than 130/85 mm Hg, HDL less than 50 mg/dL in women, or less than 40 in men or specific treatment; and fasting blood glucose greater than 100 mg/dL or antidiabetic treatment.
(b) The International Diabetes Federation (IDF) [49] requires the presence of central obesity assessed as a waist circumference greater than 80 cm in women and 94 cm in men, in addition to two of the other factors mentioned above in the ATP III requirements (triglycerides, HDL-cholesterol, blood pressure, and blood glucose).
(c) The JIS [48] model uses the same criteria as NCEP ATPIII, but with waist circumference cut-off points of 80 cm in women and 94 cm in men.

Hypertriglyceridemic waist (ATPIII and IDF criteria) [50]. The ATPIII Model requires: waist circumference >102 cm (men) and >88 cm (women) with a triglyceride level greater than 150 mg/dL or hypertriglyceridemia treatment. The IDF Model requires: waist circumference >94 cm (men) and >80 cm (women) with a triglyceride level >150 mg/dL or treatment of hypertriglyceridemia.

Hypertensive waist circumference (ATPIII and IDF criteria) [51]. ATPIII criteria include: waist circumference of 102 cm (men) and 88 cm (women) or greater, plus systolic blood pressure (SBP) greater than or equal to 130 mmHg, or Diastolic Blood Pressure (DBP) greater than or equal to 85 mmHg, or treatment for high blood pressure. The IDF criteria require: a minimum waist circumference of 94 cm in men and 80 cm in women, and at least a SBP of 130 mm Hg or a DBP of 85 mm Hg or higher, or being on antihypertensive treatment.

Atherogenic dyslipidemia was defined as triglyceride levels 150 mg/dL, HDL-c values <40 mg/dL in men and <45 mg/dL in women, and normal LDL-c. In addition, if LDL-c levels were >160, LT (lipid triad) was considered) [52].

The cardiometabolic index (CMI) was calculated using the formula TG/HDL-c× WHtR [53].

The caloric needs of the patients were estimated according to the FAO/WHO recommendations, calculating the total energy expenditure as the sum of the basal energy expenditure (BEE) or basal metabolic rate (BMR), the expenditure for physical activity (PA) and the thermogenic effect of food (TEF) [54]. To calculate basal metabolism, the resting metabolic rate (kcal/day) method was used based on weight (P) in kg and age, FAO/WHO/UNU (1985) [54] (Table 1).

The company intranet was used, in which there was a series of dietary tips prepared by the health system. This advice was personalized and based on increasing adherence to the Mediterranean diet. Each person accessed their customized report through a personal code. In addition, individualized reminders were received every 2 months via SMS on each person’s private mobile phone.

The messages have been designed by a group of experts that includes family physicians, occupational medicine physicians, nutritionists, and health education experts. The main objective of these messages is to encourage the adoption of healthy lifestyles, with special emphasis on diet.

### 2.2. Statistical Analysis

A descriptive analysis of the categorical variables was performed, calculating the frequency and distribution of the responses for each one. For quantitative variables, the mean and standard deviation were calculated, since they followed a normal distribution.

Bivariate association analysis was performed by applying the chi-squared test [2] (corrected by Fisher’s exact statistic when conditions required it) and Student’s *t*-test for independent samples (for comparison of means). Multinomial logistic regression was performed in pre- and post-intervention contexts, to assess which variables increase cardiometabolic risk. The Statistical Package for the Social Sciences (SPSS) version 28.0 (IBM Company, New York, NY, USA) for Windows was used to perform the statistical analysis, with an accepted statistical significance level of 0.05.

### 2.3. Considerations and/or Ethical Aspects

The research team ensured at all times to follow the ethical principles of research in health sciences established at the national and international level (Declaration of Helsinki). The study was approved by the Balearic Islands Research Ethics Committee (CEI-IB), with the following indicator: IB 4383/20. Participation was voluntary, with participants giving their consent to participate in the study after having received sufficient information regarding the nature of it.

The data collected for the study were identified by a code, and only the person responsible for it can relate this data to the participants. The research team strictly complied with Organic Law 3/2018, of December 5, on the protection of personal data and guarantee of digital rights, guaranteeing the participants in this study the exercise of their rights of access, rectification, cancellation, and opposition to the data collected.

## 3. Results

At the end of the confinement due to the COVID-19 pandemic, changes could be observed in both the lifestyle and health of the population, with a decrease in physical exercise and an increase in obesity [55]. This situation triggered our concern for all the people who had been harmed by this situation, which led us to consider establishing a program to encourage increased physical exercise and weight loss through computerized dietary advice, with reinforcement thereof through mobile SMS messaging, in a sample of the population of workers that we serve.

For this study, we obtained a sample of 1964 workers, 50.96% of whom were women and 49.04% men, which is a representative proportion of both sexes. The characteristics of the participants, stratified by age, physical activity, smoking, and adherence to the Mediterranean diet, are summarized in Table 1. One-third of the workers were between 40–49 years of age and were non-smokers, and more than half—64.2% of men and 57.6% of women—had higher education or held positions of responsibility (Social class I). The low adherence to the Mediterranean diet in the sample studied is noteworthy (Table 2).

In our study, we only had 1.8% of losses (36 patients), although around 20% usually occur [56]; our sample has allowed us to have very few losses, as it corresponds to people who attend work reviews.

When studying the differences between the mean values of the different cardiometabolic risk scales before and after the dietary intervention program, it stands out that, in the case of men, all the parameters improved in a statistically significant way, except for the WthipR, which obtained exactly the same results at the baseline visit and at 1 year of follow-up.

In the case of women, the behavior was different: no differences were found in the mean values of HDL-cholesterol, percentage of fat mass, or triglyceride glucose index, which remained unchanged, with no modification of the WthipR either, presenting the same behavior as their male counterparts (Table 3).

When evaluating the prevalence of high values in the different cardiometabolic risk scales before and after the dietary intervention program, we found that there was a significant improvement in all the variables evaluated in both sexes (Table 4). This table highlights that the prevalence of body fat decreased in those individuals who had a very high fat percentage, a circumstance that occurred in both sexes, but to a greater extent in women.

The pre- and post-intervention multinomial logistic regression analysis shows that, both at baseline and post-intervention, the variables that most increase the risk of presenting high values of the different cardiometabolic risk scales are physical activity and age, followed by sex, and adherence to the Mediterranean diet. Tobacco, in most cases, does not show influence (Table 5 and Table 6).

It is observed that, both at baseline and post-intervention, the variables that most increase the risk of presenting high values of the different cardiometabolic risk scales are physical activity and age, followed by sex and adherence to the Mediterranean diet. Tobacco, in many cases, shows no influence.

## 4. Discussion

This study was carried out on workers from two autonomous communities in Spain (the Balearic Islands and Valencia) who performed different professions and who went to occupational health services to undergo occupational medical examination. The intervention was carried out shortly after the confinement caused by the COVID-19 pandemic, including patients seen between the months of January and June 2021 and their follow-up for 12 months. Therefore, in the sample, it was possible to find higher basal values than those typically found under normal conditions. However, our intention was to evaluate the effectiveness of a personalized weight loss program through dietary advice prepared by the occupational health unit, in order to encourage and increase adherence to the Mediterranean diet. The usefulness of new technologies had been evidenced in other studies, which concluded that the use of the internet and interventions with mobile phones increased the consumption of fruits and vegetables by between two and four servings a day [57]. This advice was reinforced by an SMS sent to the mobile phone of each of the participants every 2 months, based on the existing literature that recommends frequent and sustained actions to obtain effective weight loss [58].

A decrease in blood pressure was obtained in both men and women, which was statistically significant in both systolic and diastolic pressure. These results are consistent with those of a previous study by Gómez-Sánchez et al. [59], who observed a drop in blood pressure with a weight loss and physical exercise program at 3 months for systolic pressure and at 12 months for both pressures. In our case, the measurement was performed only after 12 months, which suggests that, for this intervention to be effective on both components of blood pressure, it must be maintained over time, as seen in other studies [60].

We also found a decrease in BMI, waist circumference, and hip circumference that was also significant in both men and women. Similar findings were published by Lugones-Sánchez et al. in a study combining a smartphone app with a weight loss smartband [61]. In our study, we found no differences between men and women, which may be due to the fact that our sample is very similar between both sexes, whereas in the Lugones-Sánchez study, 68.5% of participants were women.

The variables that correspond to glycaemia and lipid profile also showed significant improvements in their blood values, with the exception of HDL-c figures in women, which did not show any changes. This also agrees with other obesity treatment studies, in which weight loss is associated with a reduction in LDL-c and triglycerides, but with small changes in HDL-c [62].

In the case of body fat, the average percentage of fat mass improved in men but remained unchanged in women. However, the percentage of visceral fat did show a significant decrease in both sexes. This situation had already been described in previous studies detailing that, when establishing a weight loss program, men mobilize a greater amount of intra-abdominal fat than women, which results in a more notable improvement in metabolic risk profile [63]. If we assess the prevalence of a very high percentage of body fat, we find a significant reduction in both sexes. This may suggest that the greatest loss of body fat occurred in people with the highest fat mass at the beginning of the study, which is also logical if we take into account the fact that the average BMI for women was 24.3—that is, within the figures recognized as normal weight [64].

Insulin resistance scales also showed a significant reduction between the values before and after the diet in both sexes, except for the TyG index in women, which did not present statistical significance. Similarly, the percentages of high values in the different insulin resistance formulas were also greatly decreased, between 16.2–33.3%, depending on the formula used. This indicates a significantly lower risk of suffering metabolic syndrome and consequently a decrease in the cardiometabolic risk of the individual; these are results that coincide with other studies [65,66,67].

Another cardiometabolic risk factor is NAFLD (non-alcoholic fatty liver disease), which is characterized by the accumulation of fat in the liver of people who do not consume alcohol or do so in small amounts and is associated with overweight and obesity [68]. NAFLD is an increasingly common problem in the adult population, affecting approximately 25% [69]. This can precipitate the massive accumulation of proteins in the extracellular matrix of hepatocytes, which produces liver fibrosis that can lead to cirrhosis or liver failure [70]. Its development is also favored by insulin resistance [71], which is an element that increases cardiometabolic risk.

This led to our decision to evaluate the NAFLD (LAP, FLI, HSI, FLD, ZJU) and liver fibrosis (BAAT) scales. In all of these, we found a significant improvement in both the mean values obtained at the baseline visit and at 12 months, as well as in the prevalence of high risk before and after the diet. This is of vital importance, since there are currently no drugs approved for the treatment of NAFLD, as dietary intervention and physical exercise have been shown to be effective in the treatment of NAFLD [72,73,74,75]. It is worth highlighting the values achieved in the very significant decrease in the high risk of liver fibrosis, obtained in both men and women: BAAT high of −32.4% for the former and −26.6% for the latter. This decrease in the risk of liver fibrosis, evaluated with the BAAT score, coincides with other published studies [76].

We also wanted to assess how diet monitoring influenced formulas for defining the metabolic syndrome. In Table 3, when evaluating the number of factors present in each of the formulas, the one that corresponds to the IDF was left out, since in its definition of metabolic syndrome waist circumference is a sine qua non condition [44] and would, therefore, alter the results. The other two formulas presented a significant reduction in the number of factors that make up the metabolic syndrome. When assessing the prevalence of these factors in the three formulas (Table 4), we verified that there is a very significant reduction in the prevalence of the different factors that make up the metabolic syndrome after dietary intervention, as observed in other studies [77,78,79,80]. Although this reduction was significant in both sexes, it was much more accentuated in men, with a decrease of between 42.7–39.3%, depending on the formula used, compared to 20.4–11.5% in women.

The cardiometabolic index (CMI) is an index described in 2018 to assess the distribution and dysfunction of visceral fat [52]. Several studies revealed a close relationship of this index with cardiovascular and metabolic diseases, so it should be taken into account to predict metabolic diseases [81,82,83]. In our study, we found significant differences between the results prior to the diet and those obtained in the control visit at 12 months. These results are logical when taking into consideration the fact that the CMI assesses TG, HDL-c, and the waist/height ratio, so, by obtaining an improvement in the aforementioned parameters, it is expected that the CMI will also improve. However, we believe that it is important to highlight these results, since the latest studies defend the CMI as the most significant predictor for the risk of ischemic cardiovascular disease (CVD) [84].

Atherogenic dyslipidemia and the lipid triad are two forms of alterations in the biochemistry of lipids that evolve into atherosclerosis and produce CVD [85]. In a previous study, we established the association of atherogenic dyslipidemia and the lipid triad with overweight and obesity [86], so we believe it is important to show the significant reduction that occurs in both indices after the dietary intervention program.

Finally, we have performed a pre- and post-intervention multinomial logistic regression analysis to assess which of the variables studied most increase cardiometabolic risk and if any of these are affected by dietary intervention.

The variables with the greatest influence on cardiometabolic risk are physical activity and age, in such a way that the older the person is and the less physical activity the person performs, the higher their cardiometabolic risk, which is maintained post-intervention. Other variables that also play a role are sex, with a higher cardiometabolic risk in men, and adherence to the Mediterranean diet. Lesser adherence to the Mediterranean diet correlated to greater cardiometabolic risk in all the scales used in our study.

Regarding smoking, our results show that it has no influence on most of the scales used, and that this characteristic is maintained before and after the intervention.

With regard to social class, practically all the scales used do not find differences between social class I and social class II. However, social class III does present a higher cardiometabolic risk in all the scales used.

In the comparison between pre- and post-intervention multinomial logistic regression, we observed that, in patients with good adherence to the Mediterranean diet, cardiometabolic risk decreased on all scales. In the same way, other variables, such as hypertension, obesity or metabolic syndrome, improve.

The novelty of this study is the efficacy of the prescription of dietary advice from an occupational health service, which facilitates the follow-up of patients and the messages of reinforcement in the changes in their eating habits. This creates changes in their health parameters and obtains a greater effectiveness of the health system, thus demonstrating the importance of joint work from all areas of the health system in the prevention of population health risks.

### 4.1. Limitations

One factor that could influence the results of our study is the age of the population, since more than half of our sample was between 40–59 years old. One systematic review concluded that middle-aged people have greater adherence and predisposition to the use of self-monitoring interventions [87].

Another important factor that must be taken into account is that it is a quasi-experimental study. In other words, we know that both diet and physical exercise influence weight loss [88]. In our study, we did not control for the physical exercise variable, which, as we were able to verify in the sample, was moderate to high in more than half of our population, which favors adherence to a better diet [89]. Different studies have found a strong relationship between regular physical exercise and eating habits, such that people who are more motivated to eat well have a greater tendency to do some type of physical exercise on a regular basis [90,91].

### 4.2. Strengths

However, we believe this does not detract from the validity of our results, as it is a large sample of 1964 workers, with an equal distribution between sexes, and with no loss in follow-up.

## 5. Conclusions

Our results suggest that dietary advice on a Mediterranean diet and its reinforcement with reminder messages through the use of mobile phones may be useful to improve the parameters evaluated in this study and reduce the cardiometabolic risk of patients.

It is effective to carry out a healthy eating prescription from the occupational health services, complementing and intensifying the actions of the rest of the health services.

## Figures and Tables

**Table 1:** Calculation of calorie expenditure.

Metabolic Rate at Rest Based on Weight and Age			
Age (Years)	Men	Women	
0–2	(60.9 × P) − 54	(61.0 × P) − 51	
3–9	(22.7 × P) + 495	(22.5 × P) + 499	
10–17	(17.5 × P) + 651	(12.2 × P) + 746	
18–29	(15.3 × P) + 679	(14.7 × P) + 496	
30–59	(11.6 × P) + 879	(8.7 × P) + 829	
≥60	(13.5 × P) + 487	(10.5 × P) + 596	
Total energy expenditure according to resting metabolic rate (RMR)			
Physical activity intensity	Light	Moderate	High
Men	1.55	1.78	2.10
Women	1.56	1.64	1.82
Classification of activities by intensity			
Light	People who spend several hours a day in sedentary activities, who do not regularly do sport, use the car to get around, spend most of their leisure time watching TV, reading, using the computer or video games. E.g., sitting or standing most of the time, walking on flat ground, doing light housework, board games, sewing, cooking, studying, driving, writing on a computer, office workers, etc. Those who performed light or moderate activity 2 or 3 times a week were classified in this section.		
Moderate	Walking at 5 km/h, carrying out heavy housework (cleaning windows, sweeping, etc.), carpenters, construction workers (except hard jobs), chemical and electrical industries, mechanized agricultural tasks, golf, childcare, etc. Activities in which objects are moved or handled in a moderate way. They were classified in this section if more than 30 min/day of moderate activity and up to 20 min/week of vigorous activity were carried out.		
High	People who walk long distances on a daily basis, use a bicycle to get around, carry out activities of great physical effort, or do sports that require a high level of effort for several hours. E.g., non-mechanized agricultural tasks, mining, forestry, digging, cutting firewood, mowing by hand, climbing, mountaineering, playing soccer, tennis, jogging, dancing, skiing, etc. They were classified in this section if they engaged in moderate or vigorous activity every day.		

Source: FAO/WHO-OMS/UNU Expert Consultation Report. Energy and Protein Requirements. Technical Report Series 724. Geneva: WHO/OMS. 1985 [54].

**Table 2:** Characteristics of the population.

	Men *n* = 963	Women *n* = 1001	
	%	%	*p*-Value
18–29 years	15.6	17.9	0.185
30–39 years	28.0	26.4	
40–49 years	33.0	33.0	
50–59 years	18.2	18.5	
60–69 years	5.2	4.4	
Social class I	64.2	57.6	<0.001
Social class II	11.0	13.5	
Social class III	24.8	28.9	0.490
Non-smokers	84.7	84.8	
Smokers	15.3	15.2	
Low physical exercise	25.5	33.2	<0.001
Moderate physical exercise	27.7	27.7	
High physical exercise	46.7	39.1	
Low adherence Mediterranean diet	61.2	58.2	0.113
High adherence Mediterranean diet	38.8	41.8	

**Table 3:** Mean values of different cardiometabolic risk scales before and after the dietary intervention program by sex.

	Men	*n* = 963		Women	*n* = 1001	
Dietary Intervention Program	Basal	After		Basal	After	
	Mean (SD)	Mean (SD)	*p*-Value	Mean (SD)	Mean (SD)	*p*-Value
Systolic blood pressure	129.4 (13.8)	127.7 (12.4)	<0.001	116.7 (15.0)	116.0 (12.7)	<0.001
Diastolic blood pressure	81.4 (10.9)	78.9 (9.6)	<0.001	76.6 (10.1)	73.4 (9.6)	<0.001
Glycaemia	95.0 (17.8)	92.0 (17.9)	<0.001	89.5 (11.7)	88.3 (13.3)	<0.001
Total cholesterol	194.0 (35.2)	190.3 (36.7)	<0.001	190.1 (34.7)	187.0 (34.5)	<0.001
HDL-c	47.2 (11.9)	50.0 (10.4)	<0.001	59.1 (12.9)	59.1 (12.6)	0.224
LDL-c	126.0 (30.9)	123.6 (73.6)	<0.001	115.3 (31.0)	111.5 (30.1)	<0.001
Triglycerides	117.8 (81.8)	102.7 (56.7)	<0.001	81.9 (47.7)	80.5 48.3)	<0.001
Weight	82.7 (14.8)	80.4 (14.0)	<0.001	63.9 (13.3)	63.7 (13.4)	<0.001
Waist circumference	91.8 (12.3)	88.1 (12.3)	<0.001	77.7 (12.0)	76.1 (11.7)	<0.001
Hip circumference	104.1 (8.6)	99.8 (8.2)	<0.001	101.2 (10.2)	97.6 (10.9)	<0.001
BMI	26.7 (4.3)	26.0 (4.2)	<0.001	24.3 (4.9)	24.1 (4.8)	<0.001
WtHR	0.52 (0.07)	0.50 (0.07)	<0.001	0.48 (0.08)	0.47 (0.07)	<0.001
WthipR	0.88 (0.07)	0.88 (0.07)	0.337	0.77 (0.07)	0.77 (0.08)	0.297
% Fat mass	20.1 (7.9)	19.6 (7.4)	0.01	28.9 (7.9)	28.8 (7.8)	0.084
% Visceral fat	8.2 (4.5)	7.5 (4.9)	<0.001	4.7 (3.3)	4.4 (3.1)	<0.001
TyG index	8.5 (0.6)	8.3 (0.5)	0.01	8.1 (0.5)	8.1 (0.5)	0.339
TyG-BMI index	226.6 (45.8)	217.3 (42.3)	<0.001	197.8 (47.2)	195.5 (46.4)	<0.001
TyG-waist index	779.0 (141.8)	736.7 (131.2)	<0.001	631.8 (122.8)	618.0 (119.1)	<0.001
TyG-WtHR index	4.4 (0.8)	4.2 (0.8)	<0.001	3.9 (0.8)	3.8 (0.8)	0.02
METS-IR	40.3 (9.0)	38.0 (8.2)	<0.001	33.6 (8.6)	33.1 (8.2)	<0.001
SPISE-IR	1.7 (0.5)	1.6 (0.5)	<0.001	1.4 (0.5)	1.3 (0.5)	<0.001
LAP	40.8 (44.0)	30.0 (29.4)	<0.001	20.9 (24.3)	19.4 (23.2)	<0.001
FLI	41.1 (29.4)	33.9 (29.0)	<0.001	17.6 (23.3)	15.5 (20.9)	<0.001
HSI	36.4 (6.4)	33.7 (6.5)	<0.001	36.8 (7.2)	33.8 (6.2)	<0.001
FLD	31.7 (5.4)	30.8 (5.5)	<0.001	28.1 (5.6)	27.7 (5.3)	<0.001
BAAT	1.1 (1.1)	0.8 (0.9)	<0.001	0.8 (1.0)	0.6 (0.8)	<0.001
nº factors MS ATP III	1.5 (1.4)	1.1 (1.2)	<0.001	1.0 (1.2)	0.8 (1.1)	<0.001
nº factors MS JIS	1.6 (1.4)	1.2 (1.3)	<0.001	1.2 (1.3)	1.0 (1.2)	<0.001
CMI	1.6 (1.7)	1.2 (1.0)	<0.001	0.8 (0.9)	0.7 (0.8)	<0.001

HDL-c High density lipoprotein-cholesterol. LDL-c Low density lipoprotein-cholesterol. BMI Body mass index. WtHR Waist to height ratio. WtHipR Waist to hip ratio. TyG index Trygliceride glucose index. METS-IR Metabolic score for insulin resistance.SPISE-IR single-point insulin sensitivity index. LAP Lipid accumulation product. FLI Fatty liver index. HSI Hepatic steatosis index. ZJU Zhejian University index. FLD Fatty liver disease index. MS NCEP ATPIII Metabolic syndrome National Cholesterol Education Program Adult Treatment Panel III. MS JIS Metabolic syndrome Joint Interim Societies. CMI: Cardiometabolic index.

**Table 4:** Prevalence of high values of different cardiometabolic risk scales before and after the dietary intervention program by sex.

	Men	*n* = 963			Women	*n* = 1001		
Dietary Intervention Program	Basal	After			Basal	After		
	%	%	*p*-Value	Difference %	%	%	*p*-Value	Difference %
Hypertension	25.5	21.8	<0.001	−14.5	12.1	6.7	<0.001	−44.6
Glycaemia: >100 mg/dL	29.6	18.4	<0.001	−37.8	11.7	9.5	<0.001	−18.8
Total cholesterol: high	43.3	37.7	<0.001	−12.9	38.8	32.4	<0.001	−16.5
LDL-c high	46.4	35.8	<0.001	−22.8	28.9	25.0	<0.001	−13.5
Triglycerides: high	18.7	16.2	<0.001	−13.4	6.7	5.9	<0.001	−11.9
BMI: overweight/obesity	63.2	52.0	<0.001	−17.7	35.4	32.2	<0.001	−9.0
WtHR: high	58.9	42.0	<0.001	−28.7	30.6	26.9	<0.001	−12.1
WtHipR	18.1	16.2	<0.001	−10.5	16.0	13.3	<0.001	−16.9
% Fat mass: very high	15.0	12.5	<0.001	−16.7	9.9	8.0	<0.001	−19.2
Visceral fat: high	23.7	19.0	<0.001	−19.8	3.7	2.6	<0.001	−29.7
TyG index: high	23.4	15.6	<0.001	−33.3	7.4	7.1	<0.001	−4.1
Triglycerides/HDL-c: high	38.3	32.1	<0.001	−16.2	12.9	11.7	<0.001	−9.3
METS-IR: high	14.0	9.7	<0.001	−30.7	5.7	4.7	<0.001	−17.5
SPISE-IR: high	16.8	11.5	<0.001	−31.5	6.3	5.7	<0.001	−9.5
LAP: high	28.7	20.2	<0.001	−29.6	11.3	10.2	<0.001	−9.7
FLI: high	28.4	22.5	<0.001	−20.8	8.4	6.6	<0.001	−21.4
HSI: high	44.9	39.5	<0.001	−12.0	27.8	25.6	<0.001	−7.9
ZJU: high	35.8	29.6	<0.001	−17.3	24.2	21.7	<0.001	−10.3
FLD: high	15.0	13.9	<0.001	−7.3	7.3	5.7	<0.001	−21.9
BAAT: high	31.8	21.5	<0.001	−32.4	12.8	9.4	<0.001	−26.6
MS NCEP ATPIII	21.8	12.8	<0.001	−41.3	8.7	7.7	<0.001	−11.5
MS IDF	23.4	13.4	<0.001	−42.7	10.8	8.6	<0.001	−20.4
MS JIS	26.2	15.9	<0.001	−39.3	11.1	9.3	<0.001	−16.2
Hypertriglyceridemic waist	12.8	9.0	<0.001	−29.7	5.0	4.5	<0.001	−10
Hypertensive waist	24.9	18.1	<0.001	−27.3	13.5	9.6	<0.001	−28.9
Atherogenic dyslipidemia	12.8	8.4	<0.001	−34.4	4.1	3.8	<0.001	−7.3
Lipid triad	7.8	3.4	<0.001	−56.4	2.1	1.5	<0.001	−28.6

LDL-c Low density lipoprotein-cholesterol. BMI Body mass index. WtHR Waist to height ratio. WtHipR Waist to hip ratio. TyG index Trygliceride glucose index. METS-IR Metabolic score for insulin resistance.SPISE-IR single-point insulin sensitivity index. LAP Lipid accumulation product. FLI Fatty liver index. HSI Hepatic steatosis index. ZJU Zhejian University index. FLD Fatty liver disease index. MS NCEP ATPIII Metabolic syndrome National Cholesterol Education Program Adult Treatment Panel III. MS IDF Metabolic syndrome International Diabetes Federation. MS JIS Metabolic syndrome Joint Interim Societies.

**Table 5:** Multinomial logistic regression in basal situation.

	Men	30–39 Years	40–49 Years	50–59 Years	60–69 Years	Social Class II	Social Class III	Smokers	Moderate PHE	Low PHE	Low MD
Basal	OR (95% CI)	OR (95% CI)	OR (95% CI)	OR (95% CI)	OR (95% CI)	OR (95% CI)	OR (95% CI)	OR (95% CI)	OR (95% CI)	OR (95% CI)	OR (95% CI)
Hypertension	1.8 (1.7–1.9)	1.2 (1.1–1.3)	1.5 (1.4–1.6)	2.7 (2.5–2.9)	3.9 (3.7–4.2)	ns	1.3 (1.1–1.4)	1.1 (1.0–1.2)	1.2 (1.0–1.4)	1.9 (1.7–2.1)	1.3 (1.1–1.5)
Obesity (BMI)	1.4 (1.3–1.4)	1.4 (1.3–1.6)	1.9 (1.7–2.1)	2.8 (2.6–3.1)	3.7 (3.5–3.9)	1.2 (1.1–1.4)	1.5 (1.3–1.7)	0.9 (0.9–1.0)	1.3 (1.2–1.4)	2.5 (2.1–2.9)	1.6 (1.4–1.9)
Fat mass: very high	1.7 (1.6–1.8)	1.5 (1.4–1.7)	1.8 (1.6–1.9)	2.2 (2.0–2.4)	3.1 (2.8–3.4)	1.1 (1.0–1.2)	1.4 (1.3–1.6)	1.1 (1.0–1.1)	1.5 (1.4–1.6)	3.8 (3.4–4.3)	1.7 (1.6–1.8)
Visceral fat: high	1.2 (1.2–1.3)	1.4 (1.3–1.6)	1.7 (1.6–1.9)	2.1 (2.0–2.2)	3.3 (32.1–3.6)	ns	1.5 (1.4–1.6)	1.2 (1.0–1.4)	1.7 (1.5–1.9)	3.0 (2.7–3.3)	1.9 (1.7–2.2)
TyG: high	1.9 (1.8–1.9)	1.1 (1.0–1.2)	1.4 (1.2–1.6)	1.8 (1.5–2.0)	2.2 (2.0–2.5)	1.2 (1.0–1.4)	1.6 (1.4–1.9)	ns	1.6 (1.4–1.8)	2.0 (1.7–2.2)	1.8 (1.7–1.9)
TG/HDL: high	1.8 (1.7–1.9)	1.3 (1.2–1.5)	1.6 (1.4–1.8)	2.0 (1.9–2.2)	2.5 (2.2 -2.7)	ns	1.5 (1.3–1.6)	ns	1.5 (1.4–1.7)	2.6 (2.4–2.8)	1.6 (1.4–1.7)
METS-IR: high	2.2 (2.0–2.3)	1.2 (1.0–1.3)	1.4 (1.3–1.5)	1.7 (1.6–1.9)	2.1 (1.8–2.4)	ns	1.7 (1.6–1.8)	ns	1.6 (1.4–1.8)	2.1 (2.0–2.2)	1.8 (1.7–1.8)
SPISE: high	1.7 (1.6–1.8)	ns	1.2 (1.1–1.4)	1.6 (1.5–1.8)	2.0 (1.8–2.2)	ns	1.4 (1.3–1.6)	1.1 (1.0–1.2)	1.8 (1.7–1.9)	2.4 (2.1–2.6)	1.5 (1.4–1.6)
LAP: high	1.6 (1.5–1.7)	1.2 (1.1–1.3)	1.8 (1.6–2.1)	2.0 (1.8–2.2)	2.4 (2.1–2.7)	ns	1.5 (1.3–1.7)	ns	1.9 (1.6–2.1)	2.9 (2.6–3.1)	2.0 (1.8–2.2)
FLI: high	1.4 (1.3–1.5)	ns	1.3 (1.1–1.5)	1.8 (1.5–2.1)	2.9 (2.6–3.3)	1.1 (1.0–1.2)	1.4 (1.3–1.5)	ns	1.5 (1.4–1.6)	2.8 (2.7–3.0)	1.4 (1.3–1.6)
HSI: high	1.5 (1.4–1.5)	ns	1.4 (1.3–1.6)	1.8 (1.7–1.9)	2.5 (2.3–2.7)	ns	1.7 (1.5–1.9)	ns	1.6 (1.4–1.7)	2.5 (2.3–2.7)	1.6 (1.5–1.8)
ZJU: high	1.3 (1.2–1.4)	ns	1.5 (1.4–1.7)	2.1 (1.9–2.4)	2.9 (2.6–3.1)	ns	1.4 (1.2–1.5)	ns	1.9 (1.7–2.2)	2.9 (2.8–3.0)	1.4 (1.3–1.6)
FLD: high	1.6 (1.5–1.7)	1.1 (1.0–1.2)	1.4 (1.3–1.6)	1.9 (1.7–2.2)	2.5 (2.3–2.7)	ns	1.3 (1.2–1.3)	ns	1.4 (1.2–1.6)	2.2 (2.0–2.4)	1,7 (1.5–1.9)
MS ATPIII	2.3 (2.2–2.4)	1.8 (1.6–1.9)	2.4 (2.2–2.5)	2.6 (2.5–2.8)	3.3 (3.1–3.5)	1.4 (1.2–1.6)	1.9 (1.7–2.1)	1.3 (1.1–1.4)	1.6 (1.3–1.8)	2.7 (2.4–2.9)	1.9 (1.6–2.1)
MS IDF	2.2 (2.1–2.3)	1.6 (1.4–1.8)	1.9 (1.8–2.1)	2.3 (2.1–2.6)	2.7 (2.5–3.0)	1.3 (1.1–1.5)	2.0 (1.9–2.2)	1.2 (1.0–1.3)	1.4 (1.3–1.6)	2.8 (2.7–3.0)	1.7 (1.6–1.9)
MS JIS	2.4 (2.3–2.5)	1.4 (1.3–1.6)	1.8 (1.7–2.0)	2.2 (2.0–2.5)	2.8 (2.6–3.1)	1.2 (1.1–1.4)	1.6 (1.4–1.8)	1.3 (1.2–1.3)	1.5 (1.3–1.7)	3.0 (2.8–3.3)	1.3 (1.2–1.4)
AD	2.8 (2.7–2.9)	1.3 (1.1–1.6)	1.7 (1.5–1.9)	2.2 (1.9–2.6)	2.9 (2.6–3.3)	1.3 (1.1–1.5)	1.6 (1.5–1.8)	1.4 (1.3–1.5)	1.9 (1.7–2.1)	2.9 (2.7–3.2)	1.8 (1.7–1.9)

BMI: Body mass index. TyG: Triglyceride glucose index. TG/HDL: Triglyceride/High density lipoprotein. METS-IR: Metabolic score for insulin resistance. SPISE: Single-point insulin sensitivity index LAP: Lipid accumulation product. FLI: Fatty liver index. his: Hepatic steatosis index. ZJU: Zhejiang University index. FLD: Fatty liver disease. MS: Metabolic syndrome. ATPIII: Adult Treatment Panel III. IDF: International Diabetes Federation. JIS: Joint Interim Statement. AD: Atherogenic dyslipidemia. References: Women, 18–29 years, social class I, non-smokers, high physical activity, high adherence to Mediterranean diet.

**Table 6:** Multinomial logistic regression in postintervention situation.

	Men	30–39 Years	40–49 Years	50–59 Years	60–69 Years	Social Class II	Social Class III	Smokers	Moderate PHE	Low PHE	Low MD
	OR (95% CI)	OR (95% CI)	OR (95% CI)	OR (95% CI)	OR (95% CI)	OR (95% CI)	OR (95% CI)	OR (95% CI)	OR (95% CI)	OR (95% CI)	OR (95% CI)
Hypertension	1.7 (1.5–1.9)	1.1 (1.0–1.2)	1.6 (1.5–1.7)	2.4 (2.2–2.6)	3.6 (3.4–3.8)	ns	1.3 (1.1–1.5)	1.1 (1.0–1.3)	1.3 (1.2–1.4)	2.2 (2.0–2.4)	1.6 (1.5–1.7)
Obesity (BMI)	1.5 (1.4–1.7)	1.3 (1.2–1.4)	1.8 (1.6–2.0)	2.6 (2.4–2.8)	3.5 (3.3–3.8)	1.3 (1.1–1.4)	1.6 (1.5–1.7)	0.8 (0.8–0.9)	1.4 (1.3–1.5)	3.0 (2.8–3.2)	1.9 (1.8–2.0)
Fat mass: very high	1.4 (1.3–1.4)	1.4 (1.3–1.6)	1.7 (1.6–1.8)	2.3 (2.1–2.5)	3.3 (3.0–3.5)	1.2 (1.1–1.3)	1.4 (1.3–1.5)	1.3 (1.2–1.3)	1.6 (1.5–1.7)	2.9 (2.7–3.1)	1.6 (1.5–1.7)
Visceral fat: high	1.3 (1.1–1.4)	1.3 (1.2–1.5)	1.7 (1.6–1.9)	2.4 (2.2–2.6)	3.0 (2.8–3.2)	1.1 (1.0–1.3)	1.3 (1.1–1.4)	1.2 (1.1–1.3)	1.7 (1.5–1.9)	3.4 (3.3–3.6)	1.9 (1.8–2.1)
TyG: high	1.8 (1.6–2.0)	ns	1.5 (1.4–1.6)	2.2 (2.1–2.4)	2.9 (2.8–3.0)	ns	1.2 (1.1–1.3)	ns	1.7 (1.6–1.7)	3.3 (3.1–3.5)	1.8 (1.7–1.9)
TG/HDL: high	1.7 (1.6–1.8)	1.3 (1.2–1.5)	1.5 (1.4–1.6)	2.1 (2.0–2.2)	2.7 (2.5–2.8)	1.2 (1.1–1.3)	1.7 (1.6–1.8)	ns	1.7 (1.6–1.8)	3.1 (3.0–3.2)	2.0 (1.9–2.1)
METS-IR: high	2.1 (2.0–2.3)	1.1 (1.0–1.3)	1.4 (1.3–1.5)	1.9 (1.8–2.0)	2.3 (2.2–2.5)	ns	1.4 (1.3–1.5)	1.1 (1.0–1.2)	1.5 (1.4–1.6)	2.9 (2.6–3.2)	1.7 (1.5–1.8)
SPISE: high	1.6 (1.4–1.7)	ns	1.2 (1.1–1.3)	2.2 (2.1–2.3)	2.4 (2.2–2.5)	ns	1.5 (1.4–1.6)	1.2 (1.1–1.3)	1.8 (1.6–1.9)	2.7 (2.5–2.8)	1.6 (1.4–1.8)
LAP: high	1.5 (1.4–1.6)	1.3 (1.2–1.4)	1.6 (1.5–1.7)	2.2 (2.0–2.3)	2.6 (2.5–2.8)	ns	1.3 (1.2–1.4)	ns	1.5 (1.4–1.6)	3.3 (3.2–3.4)	1.9 (1.7–2.1)
FLI: high	1.3 (1.2–1.4)	ns	1.3 (1.2–1.4)	1.9 (1.8–2.0)	2.4 (2.2–2.6)	ns	1.5 (1.4–1.7)	ns	1.6 (1.5–1.7)	3.0 (2.8–3.2)	1.8 (1.6–2.0)
HSI: high	1.5 (1.3–1.7)	ns	1.2 (1.1–1.3)	1.8 (1.7–1.9)	2.5 (2.4–2.5)	ns	1.6 (1.4–1.7)	ns	1.5 (1.4–1.6)	2.7 (2.5–2.9)	1.6 (1.5–1.7)
ZJU: high	1.4 (1.3–1.5)	ns	1.4 (1.2–1.5)	2.0 (1.8–2.1)	2.9 (2.7–3.0)	ns	1.4 (1.3–1.5)	ns	1.3 (1.2–1.3)	2.5 (2.4–2.6)	1.4 (1.3–1.5)
FLD: high	1.5 (1.4–1.6)	1.2 (1.1–1.3)	1.7 (1.6–1.9)	2.0 (1.8–2.1)	2.6 (2.4–2.8)	ns	1.3 (1.2–1.5)	ns	1.5 (1.4–1.6)	2.6 (2.4–2.8)	1.7 (1.5–1.9)
MS ATPIII	2.2 (2.1–2.4)	1.9 (1.8–2.1)	2.3 (2.1–2.5)	2.6 (2.5–2.8)	3.1 (3.0–3.2)	1.5 (1.4–1.6)	2.0 (1.8–2.1)	1.4 (1.3–1.5)	1.6 (1.5–1.8)	3.1 (3.0–3.2)	1.9 (1.8–2.1)
MS IDF	2.0 (1.9–2.1)	1.5 (1.4–1.5)	2.0 (1.8–2.1)	2.5 (2.4–2.6)	2.9 (2.8–3.0)	1.4 (1.4–1.5)	1.8 (1.7–1.9)	1.5 (1.4–1.6)	1.7 (1.6–1.8)	3.2 (3.0–3.4)	2.0 (1.9–2.1)
MS JIS	2.3 (2.2–2.5)	1.5 (1.4–1.6)	1.9 (1.7–2.0)	2.4 (2.3–2.5)	3.1 (3.0–3.3)	1.3(1.1–1.4)	1.8 (1.7–1.9)	1.4 (1.3–1.5)	1.9 (1.7–2.1)	2.9 (2.7–3.2)	1.6 (1.5–1.7)
AD	2.7 (2.6–2.8)	1.3 (1.2–1.5)	1.8 (1.6–1.9)	2.4 (2.3–2.6)	2.9 (2.7–3.0)	1.4 (1.4–1.5)	2.0 (1.8–2.1)	1.5 (1.4–1.6)	1.8 (1.5–1.7)	3.4 (3.3–3.6)	2.1 (2.0–2.1)

BMI: Body mass index. TyG: Triglyceride glucose index. TG/HDL: Triglyceride/High density lipoprotein. METS-IR: Metabolic score for insulin resistance. SPISE: Single-point insulin sensitivity index LAP: Lipid accumulation product. FLI: Fatty liver index. his: Hepatic steatosis index. ZJU: Zhejiang University index. FLD: Fatty liver disease. MS: Metabolic syndrome. ATPIII: Adult Treatment Panel III. IDF: International Diabetes Federation. JIS: Joint Interim Statement. AD: Atherogenic dyslipidemia. References: Women, 18–29 years, social class I, non-smokers, high physical activity, high adherence to Mediterranean diet.

## Data Availability

Data are available on request due to restrictions, e.g., privacy or ethical. Please contact the corresponding author.

## References

[1] World Health Organization Controlling the Global Obesity Epidemic. https://www.who.int/activities/controlling-the-global-obesity-epidemi.

[2] Centers for Disease Control (CDC) BRFSS (2021). Behavioral Risk Factor Surveillance System Survey Data.

[3] https://ourworldindata.org/causes-of-death.

[4] Bennasar-Veny M., Sergio F., López-González A., Busquets-Cortés C., Yáñez M.A. (2020). Lifestyle and progression to type 2 diabetes in a cohort of workers with prediabetes. Nutrients.

[5] Kendel Jovanović G., Mrakovcic-Sutic I., Pavičić Žeželj S., Šuša B., Rahelić D., Klobučar Majanović S. (2020). The Efficacy of an Energy-Restricted Anti-Inflammatory Diet for the Management of Obesity in Younger Adults. Nutrients.

[6] Anekwe C.V., Jarrell A.R., Townsend M.J., Gaudier G.I., Hiserodt J.M., Stanford F.C. (2020). Socioeconomics of Obesity. Curr. Obes. Rep..

[7] Ayalon I., Bodilly L., Kaplan J. (2021). The Impact of Obesity on Critical Illnesses. Shock.

[8] Zhang Z., Kahn H.S., Jackson S.L., Steele E.M., Gillespie C., Yang Q. (2022). Associations between ultra- or minimally processed food intake and three adiposity indicators among US adults: NHANES 2011 to 2016. Obesity.

[9] Verduci E., Bronsky J., Embleton N., Gerasimidis K., Indrio F., Köglmeier J., de Koning B., Lapillonne A., Moltu S.J., Norsa L. (2021). Role of Dietary Factors, Food Habits, and Lifestyle in Childhood Obesity Development: A Position Paper From the European Society for Paediatric Gastroenterology, Hepatology and Nutrition Committee on Nutrition. J. Pediatr. Gastroenterol. Nutr..

[10] Wadolowska L., Hamulka J., Kowalkowska J., Kostecka M., Wadolowska K., Biezanowska-Kopec R., Czarniecka-Skubina E., Kozirok W., Piotrowska A. (2018). Prudent-Active and Fast-Food-Sedentary Dietary-Lifestyle Patterns: The Association with Adiposity, Nutrition Knowledge and Sociodemographic Factors in Polish Teenagers-The ABC of Healthy Eating Project. Nutrients.

[11] Drozdz D., Alvarez-Pitti J., Wójcik M., Borghi C., Gabbianelli R., Mazur A., Herceg-Čavrak V., Lopez-Valcarcel B.G., Brzeziński M., Lurbe E. (2021). Obesity and Cardiometabolic Risk Factors: From Childhood to Adulthood. Nutrients.

[12] Guan W.J., Ni Z.Y., Hu Y., Liang W.H., Qu C.Q., He J.X., Liu L., Shan H., Lei C.L., Hui D.S.C. (2020). Clinical characteristics of coronavirus disease 2019 in China. N. Engl. J. Med..

[13] Wuhan Seafood Market Pneumonia Virus Isolate Wuhan-Hu-1, Complete Genome. 23 de enero de 2020 [citado 7 de 453 febrero de 2020]. http://www.ncbi.nlm.nih.gov/nuccore/MN908947.3.

[14] Koh D. (2020). COVID-19 lockdowns throughout the world. Occup. Med..

[15] Real Decreto 463/2020, de 14 de Marzo, por el que se Declara el Estado de Alarma Para la Gestión de la Situación de Crisis 461 Sanitaria Ocasionada por el COVID-19. https://www.boe.es/buscar/doc.php?id=BOE-A-2020-3692.

[16] Ramírez Manent J.I., Altisench Jané B., Sanchís Cortés P., Busquets-Cortés C., Arroyo Bote S., Masmiquel Comas L., López González Á.A. (2022). Impact of COVID-19 Lockdown on Anthropometric Variables, Blood Pressure, and Glucose and Lipid Profile in Healthy Adults: A before and after Pandemic Lockdown Longitudinal Study. Nutrients.

[17] Wiechert M., Holzapfel C. (2021). Nutrition Concepts for the Treatment of Obesity in Adults. Nutrients.

[18] Chao A.M., Quigley K.M., Wadden T.A. (2021). Dietary interventions for obesity: Clinical and mechanistic findings. J. Clin. Investig..

[19] Estruch R., Ros E., Salas-Salvadó J., Covas M.-I., Corella D., Arós F., Gómez-Gracia E., Ruiz-Gutiérrez V., Fiol M., Lapetra J. (2018). PREDIMED Study Investigators. Primary Prevention of Cardiovascular Disease with a Mediterranean Diet Supplemented with Extra-Virgin Olive Oil or Nuts. N. Engl. J. Med..

[20] Davis C., Bryan J., Hodgson J., Murphy K. (2015). Definition of the Mediterranean Diet: A Literature Review. Nutrients.

[21] https://estilosdevidasaludable.sanidad.gob.es/.

[22] Cavero-Redondo I., Martinez-Vizcaino V., Fernandez-Rodriguez R., Saz-Lara A., Pascual-Morena C., Álvarez-Bueno C. (2020). Effect of Behavioral Weight Management Interventions Using Lifestyle mHealth Self-Monitoring on Weight Loss: A Systematic Review and Meta-Analysis. Nutrients.

[23] Burke L.E., Sereika S.M., Bizhanova Z., Parmanto B., Kariuki J., Cheng J., Beatrice B., Cedillo M., Loar I., Pulantara I.W. (2022). The Effect of Tailored, Daily, Smartphone Feedback to Lifestyle Self-Monitoring on Weight Loss at 12 Months: The SMARTER Randomized Clinical Trial. J. Med. Internet Res..

[24] Rumbo-Rodríguez L., Sánchez-SanSegundo M., Ruiz-Robledillo N., Albaladejo-Blázquez N., Ferrer-Cascales R., Zaragoza-Martí A. (2020). Use of Technology-Based Interventions in the Treatment of Patients with Overweight and Obesity: A Systematic Review. Nutrients.

[25] Phillips N., Mareschal J., Schwab N., Manoogian E., Borloz S., Ostinelli G., Gauthier-Jaques A., Umwali S., Rodriguez E.G., Aeberli D. (2021). The Effects of Time-Restricted Eating versus Standard Dietary Advice on Weight, Metabolic Health and the Consumption of Processed Food: A Pragmatic Randomised Controlled Trial in Community-Based Adults. Nutrients.

[26] Free C., Phillips G., Galli L., Watson L., Felix L., Edwards P., Patel V., Haines A. (2013). The effectiveness of mobile-health technology-based health behaviour change or disease management interventions for health care consumers: A systematic review. PLoS Med..

[27] Fischer H.H., Fischer I.P., Pereira R.I., Furniss A.L., Rozwadowski J.M., Moore S.L., Durfee M.J., Raghunath S.G., Tsai A.G., Havranek E.P. (2016). Text Message Support for Weight Loss in Patients With Prediabetes: A Randomized Clinical Trial. Diabetes Care.

[28] Bhardwaj N.N., Wodajo B., Gochipathala K., Paul D.P., Coustasse A. (2017). Can mHealth Revolutionize the Way We Manage Adult Obesity?. Perspect. Health Inf. Manag..

[29] Wang Y., Min J., Khuri J., Xue H., Xie B., AKaminsky L., JCheskin L. (2020). Effectiveness of Mobile Health Interventions on Diabetes and Obesity Treatment and Management: Systematic Review of Systematic Reviews. JMIR Mhealth Uhealth.

[30] Arambepola C., Ricci-Cabello I., Manikavasagam P., Roberts N., French D.P., Farmer A. (2016). The Impact of Automated Brief Messages Promoting Lifestyle Changes Delivered Via Mobile Devices to People with Type 2 Diabetes: A Systematic Literature Review and Meta-Analysis of Controlled Trials. J. Med. Internet Res..

[31] Schoeppe S., Alley S., Van Lippevelde W., Bray N.A., Williams S.L., Duncan M.J., Vandelanotte C. (2016). Efficacy of interventions that use apps to improve diet, physical activity and sedentary behaviour: A systematic review. Int. J. Behav. Nutr. Phys. Act..

[32] Spring B., Pellegrini C., McFadden H.G., Pfammatter A.F., Stump T.K., Siddique J., King A.C., Hedeker D. (2018). Multicomponent mHealth Intervention for Large, Sustained Change in Multiple Diet and Activity Risk Behaviors: The Make Better Choices 2 Randomized Controlled Trial. J. Med. Internet Res..

[33] Bian R.R., Piatt G.A., Sen A., Plegue M.A., De Michele M.L., Hafez D., Czuhajewski C.M., Buis L.R., Kaufman N., Richardson C. (2017). The Effect of Technology-Mediated Diabetes Prevention Interventions on Weight: A Meta-Analysis. J. Med. Internet Res..

[34] Tsaban G., Meir A.Y., Rinott E., Zelicha H., Kaplan A., Shalev A., Katz A., Rudich A., Tirosh A., Shelef I. (2020). The effect of green Mediterranean diet on cardiometabolic risk; a randomised controlled trial. Heart.

[35] Schuppelius B., Peters B., Ottawa A., Pivovarova-Ramich O. (2021). Time Restricted Eating: A Dietary Strategy to Prevent and Treat Metabolic Disturbances. Front. Endocrinol..

[36] Gepner Y., Shelef I., Komy O., Cohen N., Schwarzfuchs D., Bril N., Rein M., Serfaty D., Kenigsbuch S., Zelicha H. (2019). The beneficial effects of Mediterranean diet over low-fat diet may be mediated by decreasing hepatic fat content. J. Hepatol..

[37] Domingo-Salvany A., Bacigalupe A., Carrasco J.M., Espelt A., Ferrando J., Borrell C. (2013). del Grupo de Determinantes Sociales de Sociedad Española de Epidemiología. Propuestas de clase social neoweberiana y neomarxista a partir de la Clasificación Nacional de Ocupaciones 2011 [Proposals for social class classification based on the Spanish National Classification of Occupations 2011 using neo-Weberian and neo-Marxist approaches]. Gac. Sanit..

[38] Lee P.H., Macfarlane D.J., Lam T.H., Stewart S.M. (2011). Validity of the International Physical Activity Questionnaire Short Form (IPAQ-SF): A systematic review. Int. J. Behav. Nutr. Phys. Act..

[39] Miró O., Martín-Sánchez F.J., Jacob J., Andueza J.A., Herrero P., Llorens P. (2016). Valoración del grado de adherencia a la dieta mediterránea en pacientes con insuficiencia cardiaca: Estudio DIME-EAHFE [Evaluation of the degree of adherence to the Mediterranean diet in patients with heart failure: DIME-EAHFE study]. An. Sist. Sanit. Navar..

[40] Stewart A., Marfell-Jones M., Olds T., Ridder H. (2011). International Standards for Anthropometric Assessment.

[41] López-González A.A., Ramírez Manent J.I., Vicente-Herrero M.T., García Ruiz E., Albaladejo Blanco M., López Safont N. (2022). Prevalence of diabesity in the Spanish working population: Influence of sociodemographic variables and tobacco consumption. An. Sist. Sanit. Navar..

[42] Mohebbi V., Aramayo A., Morales J. (2021). Determination of scales related to cardiovascular risk and fatty liver in 5.370 spanish farmers. Med. Balear..

[43] Barchetta I., Dule S., Bertoccini L., Cimini F.A., Sentinelli F., Bailetti D., Marini G., Barbonetti A., Loche S., Cossu E. (2022). The single-point insulin sensitivity estimator (SPISE) index is a strong predictor of abnormal glucose metabolism in overweight/obese children: A long-term follow-up study. J. Endocrinol. Investig..

[44] Shi F., Leng J., Cao W., Tan Z., Meng W., Wang S., Xu Y. (2013). Fatty liver disease index: A simple screening tool to facilitate diagnosis of nonalcoholic fatty liver disease in the Chinese population. Dig. Dis. Sci..

[45] Wang J., Xu C., Xun Y., Lu Z., Shi J., Yu C., Li Y. (2015). ZJU index: A novel model for predicting nonalcoholic fatty liver disease in a Chinese population. Sci. Rep..

[46] Ratziu V., Giral P., Charlotte F., Bruckert E., Thibault V., Theodorou I., Khalil L., Turpin G., Opolon P., Poynard T. (2000). Liver fibrosis in overweight patients. Gastroenterology.

[47] Burton R.F. (2020). The waist-hip ratio: A flawed index. Ann. Hum. Biol..

[48] Cabrera-Rode E., Stusser B., Cálix W., Orlandi N., Rodríguez J., Cubas-Dueñas I., Echevarría R., Álvarez A. (2017). Concordancia diagnóstica entre siete definiciones de síndrome metabólico en adultos con sobrepeso y obesidad [Diagnostic concordance between seven definitions of metabolic syndrome in overweight and obese adults]. Rev. Peru. Med. Exp. Salud Publica.

[49] Zimmet P., Alberti G., Serrano Ríos M. (2005). Una nueva definición mundial del síndrome metabólico propuesta por la federación Internacional de Diabetes: Fundamento y resultados [A new international diabetes federation worldwide definition of the metabolic syndrome: The rationale and the results]. Rev. Esp. Cardiol..

[50] Sam S., Haffner S., Davidson M.H., D’Agostino RBSr Feinstein S., Kondos G., Perez A., Mazzone T. (2009). Hypertriglyceridemic waist phenotype predicts increased visceral fat in subjects with type 2 diabetes. Diabetes Care.

[51] Nita C., Hancu N., Rusu A., Bala C., Roman G. (2009). Hypertensive waist: First step of the screening for metabolic syndrome. Metab. Syndr. Relat. Disord..

[52] Ponte-Negretti C.I., Isea-Perez J.E., Lorenzatti A.J., Lopez-Jaramillo P., Wyss Q.F.S., Pintó X. (2017). Atherogenic Dyslipidemia in Latin America: Prevalence, causes and treatment: Expert’s position paper made by The Latin American Academy for the Study of Lipids (ALALIP) Endorsed by the Inter-American Society of Cardiology (IASC), the South American Society of Cardiology (SSC), the Pan-American College of Endothelium (PACE), and the International Atherosclerosis Society (IAS). Int. J. Cardiol..

[53] Shi W.R., Wang H.Y., Chen S., Guo X.F., Li Z., Sun Y.X. (2018). Estimate of prevalent diabetes from cardiometabolic index in general Chinese population: A community-based study. Lipids Health Dis..

[54] World Health Organization Joint FAO/WHO/UNU Consultative Meeting of Experts on Energy and Protein Requirements (1981: Rome, Italy), Food and Agriculture Organization of the United Nations, World Health Organization & United Nations University. (1985). Energy and protein requirements: Report of a Joint FAO/WHO/UNU Consultative Meeting of Experts, [Rome, 5–17 October 1981]. https://apps.who.int/iris/handle/10665/40157.

[55] López-González Á.A., Altisench Jané B., Masmiquel Comas L., Arroyo Bote S., González San Miguel H.M., Ramírez Manent J.I. (2022). Impact of COVID-19 Lockdown on Non-Alcoholic Fatty Liver Disease and Insulin Resistance in Adults: A before and after Pandemic Lockdown Longitudinal Study. Nutrients.

[56] Hincapie Tabares D., Perez Carrillo V., Donado Gómez J.H. (2019). Causas de Pérdidas de Pacientes Durante los Ensayos Clínicos con Asignación Aleatoria: Estudio Metaepidemiológico.

[57] Afshin A., Babalola D., Mclean M., Yu Z., Ma W., Chen C.-Y., Arabi M., Mozaffarian D. (2016). Information Technology and Lifestyle: A Systematic Evaluation of Internet and Mobile Interventions for Improving Diet, Physical Activity, Obesity, Tobacco, and Alcohol Use. J. Am. Heart Assoc..

[58] Singh N., Stewart R.A.H., Benatar J.R. (2019). Intensity and duration of lifestyle interventions for long-term weight loss and association with mortality: A meta-analysis of randomised trials. BMJ Open.

[59] Gómez-Sánchez L., Gómez-Sánchez M., Lugones-Sánchez C., Rodríguez-Sánchez E., Tamayo-Morales O., Gonzalez-Sánchez S., Magallón-Botaya R., Ramirez-Manent J.I., Recio-Rodriguez J.I., Agudo-Conde C. (2022). Long-Term Effectiveness of a Smartphone App and a Smart Band on Arterial Stiffness and Central Hemodynamic Parameters in a Population with Overweight and Obesity (Evident 3 Study): Randomised Controlled Trial. Nutrients.

[60] Leslie W.S., Ali E., Harris L., Messow C.M., Brosnahan N.T., Thom G., McCombie E.L., Barnes A.C., Sattar N., Taylor R. (2021). Antihypertensive medication needs and blood pressure control with weight loss in the Diabetes Remission Clinical Trial (DiRECT). Diabetologia.

[61] Lugones-Sanchez C., Recio-Rodriguez J.I., Agudo-Conde C., Repiso-Gento I., Adalia E.G., Ramirez-Manent J.I., Sanchez-Calavera M.A., Rodriguez-Sanchez E., Gomez-Marcos M.A., Garcia-Ortiz L. (2022). EVIDENT 3 Investigators. Long-term Effectiveness of a Smartphone App Combined With a Smart Band on Weight Loss, Physical Activity, and Caloric Intake in a Population With Overweight and Obesity (Evident 3 Study): Randomized Controlled Trial. J. Med. Internet Res..

[62] Stanek A., Brożyna-Tkaczyk K., Zolghadri S., Cholewka A., Myśliński W. (2022). The Role of Intermittent Energy Restriction Diet on Metabolic Profile and Weight Loss among Obese Adults. Nutrients.

[63] Christensen P., Larsen T.M., Westerterp-Plantenga M., Macdonald I., Martinez J.A., Handjiev S., Poppitt S., Hansen S., Ritz C., Astrup A. (2018). Men and women respond differently to rapid weight loss: Metabolic outcomes of a multi-centre intervention study after a low-energy diet in 2500 overweight, individuals with pre-diabetes (PREVIEW). Diabetes Obes. Metab..

[64] Cooper A.J., Gupta S.R., Moustafa A.F., Chao A.M. (2021). Sex/Gender Differences in Obesity Prevalence, Comorbidities, and Treatment. Curr. Obes. Rep..

[65] Mirabelli M., Chiefari E., Arcidiacono B., Corigliano D.M., Brunetti F.S., Maggisano V., Russo D., Foti D.P., Brunetti A. (2020). Mediterranean Diet Nutrients to Turn the Tide against Insulin Resistance and Related Diseases. Nutrients.

[66] Tricò D., Moriconi D., Berta R., Baldi S., Quinones-Galvan A., Guiducci L., Taddei S., Mari A., Nannipieri M. (2021). Effects of Low-Carbohydrate versus Mediterranean Diets on Weight Loss, Glucose Metabolism, Insulin Kinetics and β-Cell Function in Morbidly Obese Individuals. Nutrients.

[67] Dellis D., Tsilingiris D., Eleftheriadou I., Tentolouris A., Sfikakis P.P., Dellis G., Karanasiou M., Meimari A., Dimosthenopoulos C., Lazarou S. (2020). Carbohydrate restriction in the morning increases weight loss effect of a hypocaloric Mediterranean type diet: A randomized, parallel group dietary intervention in overweight and obese subjects. Nutrition.

[68] Fujii H., Kawada N., Japan Study Group Of Nafld Jsg-Nafld (2020). The Role of Insulin Resistance and Diabetes in Nonalcoholic Fatty Liver Disease. Int. J. Mol. Sci..

[69] Pimpin L., Cortez-Pinto H., Negro F., Corbould E., Lazarus J.V., Webber L., Sheron N. (2018). EASL HEPAHEALTH Steering Committee. Burden of liver disease in Europe: Epidemiology and analysis of risk factors to identify prevention policies. J. Hepatol..

[70] Roehlen N., Crouchet E., Baumert T.F. (2020). Liver Fibrosis: Mechanistic Concepts and Therapeutic Perspectives. Cells.

[71] Ramírez-Manent J.I., Martínez-Almoyna E., López C., Busquets-Cortés C., González San Miguel H., López-González Á.A. (2022). Relationship between Insulin Resistance Risk Scales and Non-Alcoholic Fatty Liver Disease and Liver Fibrosis Scales in 219,477 Spanish Workers. Metabolites.

[72] Gao Y., Zhang W., Zeng L.Q., Bai H., Li J., Zhou J., Zhou G.Y., Fang C.W., Wang F., Qin X.J. (2020). Exercise and dietary intervention ameliorate high-fat diet-induced NAFLD and liver aging by inducing lipophagy. Redox Biol..

[73] Monserrat-Mesquida M., Quetglas-Llabrés M., Bouzas C., Montemayor S., Mascaró C.M., Casares M., Llompart I., Ugarriza L., Martínez J.A., Tur J.A. (2022). Increased Adherence to the Mediterranean Diet after Lifestyle Intervention Improves Oxidative and Inflammatory Status in Patients with Non-Alcoholic Fatty Liver Disease. Antioxidants.

[74] Cai H., Qin Y.-L., Shi Z.-Y., Chen J.-H., Zeng M.-J., Zhou W., Chen R.-Q., Chen Z.-Y. (2019). Effects of alternate-day fasting on body weight and dyslipidaemia in patients with non-alcoholic fatty liver disease: A randomised controlled trial. BMC Gastroenterol..

[75] Meir A.Y., Rinott E., Tsaban G., Zelicha H., Kaplan A., Rosen P., Shelef I., Youngster I., Shalev A., Blüher M. (2021). Effect of green-Mediterranean diet on intrahepatic fat: The DIRECT PLUS randomised controlled trial. Gut.

[76] Cantero I., Abete I., del Bas J.M., Caimari A., Arola L., Zulet M.A., Martinez J.A. (2018). Changes in lysophospholipids and liver status after weight loss: The RESMENA study. Nutr. Metab..

[77] Gershuni V.M., Yan S.L., Medici V. (2018). Nutritional Ketosis for Weight Management and Reversal of Metabolic Syndrome. Curr. Nutr. Rep..

[78] Hyde P.N., Sapper T.N., Crabtree C.D., LaFountain R.A., Bowling M.L., Buga A., Fell B., McSwiney F.T., Dickerson R.M., Miller V.J. (2019). Dietary carbohydrate restriction improves metabolic syndrome independent of weight loss. JCI Insight.

[79] Willems A.E.M., Sura-de Jong M., van Beek A.P., Nederhof E., van Dijk G. (2021). Effects of macronutrient intake in obesity: A meta-analysis of low-carbohydrate and low-fat diets on markers of the metabolic syndrome. Nutr. Rev..

[80] Sundfør T.M., Svendsen M., Tonstad S. (2018). Effect of intermittent versus continuous energy restriction on weight loss, maintenance and cardiometabolic risk: A randomized 1-year trial. Nutr. Metab. Cardiovasc. Dis..

[81] Dursun M., Besiroglu H., Otunctemur A., Ozbek E. (2016). Association between cardiometabolic index and erectile dysfunction: A new index for predicting cardiovascular disease. Kaohsiung J. Med. Sci..

[82] Wang H., Chen Y., Sun G., Jia P., Qian H., Sun Y. (2018). Validity of cardiometabolic index, lipid accumulation product, and body adiposity index in predicting the risk of hypertension in Chinese population. Postgrad Med..

[83] Zou J., Xiong H., Zhang H., Hu C., Lu S., Zou Y. (2022). Association between the cardiometabolic index and non-alcoholic fatty liver disease: Insights from a general population. BMC Gastroenterol..

[84] Higashiyama A., Wakabayashi I., Okamura T., Kokubo Y., Watanabe M., Takegami M., Honda-Kohmo K., Okayama A., Miyamoto Y. (2021). The Risk of Fasting Triglycerides and its Related Indices for Ischemic Cardiovascular Diseases in Japanese Community Dwellers: The Suita Study. J. Atheroscler. Thromb..

[85] Ascaso J.F., Millán J., Hernández-Mijares A., Blasco M., Brea Á., Díaz Á., Pedro-Botet J., Pintó X. (2020). Atherogenic Dyslipidaemia 2019. Consensus document of the Atherogenic Dyslipidaemia Group of the Spanish Arteriosclerosis Society. Clin. Investig. Arterioscler..

[86] Busquets-Cortés C., López C., Paublini H., Arroyo Bote S., López-González Á.A., Ramírez-Manent J.I. (2022). Relationship between Atherogenic Dyslipidaemia and Lipid Triad with Different Scales of Overweight and Obesity in 418,343 Spanish Workers. J. Nutr. Metab..

[87] Cheatham S.W., Stull K.R., Fantigrassi M., Motel I. (2018). The efficacy of wearable activity tracking technology as part of a weight loss program: A systematic review. J. Sports Med. Phys. Fit..

[88] Thivel D., Doucet E., Julian V., Cardenoux C., Boirie Y., Duclos M. (2017). Nutritional compensation to exercise- vs. diet-induced acute energy deficit in adolescents with obesity. Physiol. Behav..

[89] Danielsen K.K., Svendsen M., Mæhlum S., Sundgot-Borgen J. (2013). Changes in body composition, cardiovascular disease risk factors, and eating behavior after an intensive lifestyle intervention with high volume of physical activity in severely obese subjects: A prospective clinical controlled trial. J. Obes..

[90] Gast J., Campbell Nielson A., Hunt A., Leiker J.J. (2015). Intuitive eating: Associations with physical activity motivation and BMI. Am. J. Health Promot..

[91] Castro E.A., Carraça E.V., Cupeiro R., López-Plaza B., Teixeira P.J., González-Lamuño D., Peinado A.B. (2020). The Effects of the Type of Exercise and Physical Activity on Eating Behavior and Body Composition in Overweight and Obese Subjects. Nutrients.

